# 5-(3,6-Dibromo-9*H*-carbazol-9-yl)penta­nenitrile

**DOI:** 10.1107/S1600536811005162

**Published:** 2011-02-16

**Authors:** Nesimi Uludağ, Murat Ateş, Barış Tercan, Tuncer Hökelek

**Affiliations:** a Department of Chemistry, Faculty of Arts and Sciences, Namık Kemal University, 59030 Değirmenaltı, Tekirdağ, Turkey; b Department of Physics, Karabük University, 78050, Karabük, Turkey; c Department of Physics, Hacettepe University, 06800 Beytepe, Ankara, Turkey

## Abstract

In the title compound, C_17_H_14_Br_2_N_2_, the carbazole skeleton is nearly planar [maximum deviation = 0.055 (2) Å]. In the crystal, aromatic π–π stacking is observed between parallel carbazole ring systems of adjacent mol­ecules, the shortest centroid–centroid distance between benzene rings being 3.4769 (11) Å.

## Related literature

For tetra­hydro­carbazole systems present in the framework of a number of indole-type alkaloids of biological inter­est, see: Saxton (1983 ▶). For related structures and background references, see: Patır *et al.* (1997 ▶); Hökelek & Patır (1999 ▶). For applications of carbazole derivatives, see: Cloutet *et al.* (1999 ▶); Wei *et al.* (2006 ▶); Tirapattur *et al.* (2003 ▶); Taoudi *et al.* (2001 ▶); Saraswathi *et al.* (1999 ▶); Sarac *et al.* (2000 ▶).
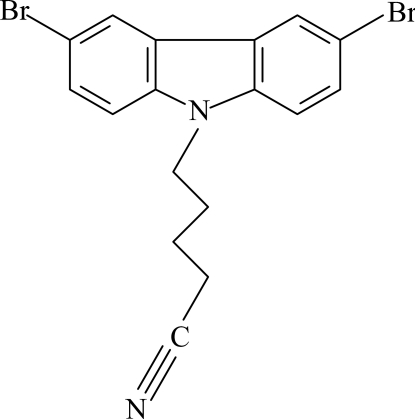


## Experimental

### 

#### Crystal data

C_17_H_14_Br_2_N_2_
*M* *_r_* = 406.10
Monoclinic, 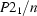

*a* = 10.5654 (2) Å
*b* = 13.1471 (3) Å
*c* = 11.6260 (2) Å
β = 105.257 (2)°
*V* = 1557.99 (6) Å^3^
*Z* = 4
Mo *K*α radiation
μ = 5.20 mm^−1^
*T* = 100 K
0.34 × 0.27 × 0.24 mm

#### Data collection

Bruker Kappa APEXII CCD area-detector diffractometer
Absorption correction: multi-scan (*SADABS*; Bruker, 2005 ▶) *T*_min_ = 0.201, *T*_max_ = 0.286
15409 measured reflections
3906 independent reflections
3344 reflections with *I* > 2σ(*I*)
*R*_int_ = 0.022

#### Refinement

*R*[*F* ^2^ > 2σ(*F* ^2^)] = 0.023
*wR*(*F* ^2^) = 0.054
*S* = 1.04
3906 reflections
190 parameters
H-atom parameters constrained
Δρ_max_ = 0.52 e Å^−3^
Δρ_min_ = −0.36 e Å^−3^

### 

Data collection: *APEX2* (Bruker, 2007 ▶); cell refinement: *SAINT* (Bruker, 2007 ▶); data reduction: *SAINT*; program(s) used to solve structure: *SHELXS97* (Sheldrick, 2008 ▶); program(s) used to refine structure: *SHELXL97* (Sheldrick, 2008 ▶); molecular graphics: *ORTEP-3 for Windows* (Farrugia, 1997 ▶); software used to prepare material for publication: *WinGX* (Farrugia, 1999 ▶) and *PLATON* (Spek, 2009 ▶).

## Supplementary Material

Crystal structure: contains datablocks I, global. DOI: 10.1107/S1600536811005162/xu5160sup1.cif

Structure factors: contains datablocks I. DOI: 10.1107/S1600536811005162/xu5160Isup2.hkl

Additional supplementary materials:  crystallographic information; 3D view; checkCIF report

## Figures and Tables

**Fig. 1.**
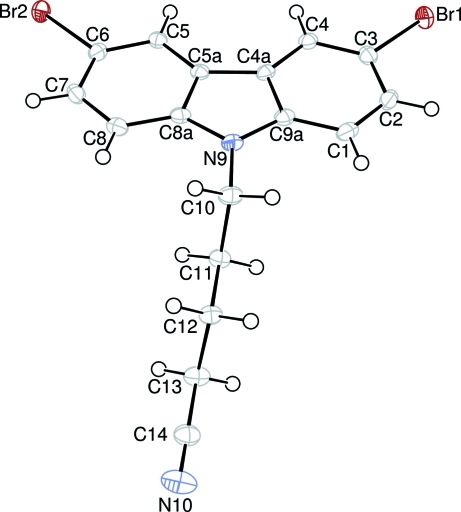
The molecular structure of the title molecule with the atom-numbering scheme. The displacement ellipsoids are drawn at the 50% probability level.

**Fig. 2.**
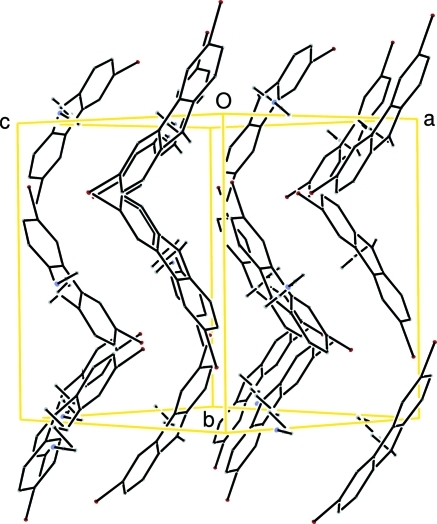
A partial packing diagram. Hydrogen atoms have been omitted for clarity.

## supplementary crystallographic information

### Comment

Tetrahydrocarbazole systems are present in the framework of a number of indole-type alkaloids of biological interest (Saxton, 1983). The structures of tricyclic, tetracyclic and pentacyclic ring systems with dithiolane and other substituents of the tetrahydrocarbazole core, have been reported previously (Patır *et al.*, 1997; Hökelek & Patır, 1999). Substituted carbazole based monomers exhibit good electroactive and photoactive properties which make them the most promising candidates for hole transporting mobility of charge carriers (Cloutet *et al.*, 1999) and photoluminescence efficiencies (Wei *et al.*, 2006). Carbazole based heterocyclic polymer systems can be chemically or electrochemically polymerized to yield materials with interesting properties with a number of applications, such as electroluminescent (Tirapattur *et al.*, 2003), photoactive devices (Taoudi *et al.*, 2001), sensors and rechargable batteries (Saraswathi *et al.*, 1999) and electrochromic displays (Sarac *et al.*, 2000). The title compound, (I), may be considered as a synthetic precursor of tetracyclic indole alkaloids of biological interests. The present study was undertaken to ascertain its crystal structure.

The title compound consists of a carbazole skeleton with a pentanenitrile group (Fig. 1), where the bond lengths and angles are within normal ranges, and generally agree with those in the previously reported compounds. In all structures atom N9 is substituted.

An examination of the deviations from the least-squares planes through individual rings shows that rings A (C1—C4/C4a/C9a), B (C4a/C5a/C8a/N9/C9a) and C (C5a/C5—C8/C8a) are planar. The carbazole skeleton, containing the rings A, B and C is also nearly coplanar [with a maximum deviation of 0.055 (2) Å for atom C2] with dihedral angles of A/B = 2.10 (6), A/C = 2.79 (5) and B/C = 0.69 (5) °. Atoms Br1, C10 and Br2 displaced by 0.0476 (2), 0.062 (2) and 0.0052 (2) Å from the corresponding planes of the carbazole skeleton.

In the crystal structure, molecules are alongated along the *b* axis and stacked nearly parallel to (101) (Fig. 2). The π···π contacts between the pyrrole and benzene rings and the benzene rings, *Cg*2—*Cg*3^i^ and *Cg*3···*Cg*3^i^ [symmetry code: (i) -*x*, 1 - *y*, -*z*, where *Cg*1, *Cg*2 and *Cg*3 are centroids of the rings A (C1—C4/C4a/C9a), B (C4a/C5a/C8a/N9/C9a) and C (C5a/C5—C8/C8a), respectively] may stabilize the structure, with centroid-centroid distances of 3.548 (1) and 3.4769 (11) Å, respectively.

### Experimental

For the preparation of the title compound, (I), sodium hydride (1.16 g, 30.76 mmol) was added to a solution of 3,6-dibromocarbazole (5.00 g, 15.38 mmol) in dry tetrahydrofuran (200 ml) in several portions, and stirred at 353 K for 2 h under argon atmosphere. Then, chlorovaleronitrile (3.46 ml, 30.76 mmol) was added and stirred at 373 K for 6 d. The reaction mixture was cooled in an ice bath, and hydrochloric acid (10%, 200 ml) was added. After the extraction with chloroform (300 ml), the organic layer was dried over anhydrous magnesium sulfate and the solvent was evaporated under reduced pressure. The residue was purified by column chromatography using silica gel and chloroform, and the product was recrystallized from diethyl ether (yield 4.50 g, 80.12%; m.p. 327 K).

### Refinement

H atoms were positioned geometrically with C—H = 0.95 and 0.99 Å for aromatic and methylene H atoms, respectively, and constrained to ride on their parent atoms, with *U*_iso_(H) = *1.2U*_eq_(C).

### Crystal data

**Table:**

C_17_H_14_Br_2_N_2_	*F*(000) = 800
*M**_r_* = 406.10	*D*_x_ = 1.731 Mg m^−^^3^
Monoclinic, *P*2_1_/*n*	Mo *K*α radiation, λ = 0.71073 Å
Hall symbol: -P 2yn	Cell parameters from 6957 reflections
*a* = 10.5654 (2) Å	θ = 2.3–28.3°
*b* = 13.1471 (3) Å	µ = 5.20 mm^−^^1^
*c* = 11.6260 (2) Å	*T* = 100 K
β = 105.257 (2)°	Block, colorless
*V* = 1557.99 (6) Å^3^	0.34 × 0.27 × 0.24 mm
*Z* = 4	

### Data collection

**Table:**

Bruker Kappa APEXII CCD area-detector diffractometer	3906 independent reflections
Radiation source: fine-focus sealed tube	3344 reflections with *I* > 2σ(*I*)
graphite	*R*_int_ = 0.022
φ and ω scans	θ_max_ = 28.4°, θ_min_ = 2.3°
Absorption correction: multi-scan (*SADABS*; Bruker, 2005)	*h* = −14→12
*T*_min_ = 0.201, *T*_max_ = 0.286	*k* = −17→16
15409 measured reflections	*l* = −15→15

### Refinement

**Table:**

Refinement on *F*^2^	Primary atom site location: structure-invariant direct methods
Least-squares matrix: full	Secondary atom site location: difference Fourier map
*R*[*F*^2^ > 2σ(*F*^2^)] = 0.023	Hydrogen site location: inferred from neighbouring sites
*wR*(*F*^2^) = 0.054	H-atom parameters constrained
*S* = 1.04	*w* = 1/[σ^2^(*F*_o_^2^) + (0.0246*P*)^2^ + 0.8394*P*] where *P* = (*F*_o_^2^ + 2*F*_c_^2^)/3
3906 reflections	(Δ/σ)_max_ = 0.001
190 parameters	Δρ_max_ = 0.52 e Å^−^^3^
0 restraints	Δρ_min_ = −0.36 e Å^−^^3^

### Special details

**Table:**

Geometry. All e.s.d.'s (except the e.s.d. in the dihedral angle between two l.s. planes) are estimated using the full covariance matrix. The cell e.s.d.'s are taken into account individually in the estimation of e.s.d.'s in distances, angles and torsion angles; correlations between e.s.d.'s in cell parameters are only used when they are defined by crystal symmetry. An approximate (isotropic) treatment of cell e.s.d.'s is used for estimating e.s.d.'s involving l.s. planes.
Refinement. Refinement of *F*^2^ against ALL reflections. The weighted *R*-factor *wR* and goodness of fit *S* are based on *F*^2^, conventional *R*-factors *R* are based on *F*, with *F* set to zero for negative *F*^2^. The threshold expression of *F*^2^ > σ(*F*^2^) is used only for calculating *R*-factors(gt) *etc*. and is not relevant to the choice of reflections for refinement. *R*-factors based on *F*^2^ are statistically about twice as large as those based on *F*, and *R*- factors based on ALL data will be even larger.

### Fractional atomic coordinates and isotropic or equivalent isotropic displacement parameters (Å^2^)

**Table:**

	*x*	*y*	*z*	*U*_iso_*/*U*_eq_	
Br1	1.00971 (2)	0.746932 (15)	0.392293 (17)	0.02544 (6)	
Br2	0.636774 (19)	1.287581 (14)	0.589087 (17)	0.02193 (6)	
C1	0.6131 (2)	0.78706 (15)	0.22924 (16)	0.0211 (4)	
H1	0.5468	0.7526	0.1712	0.025*	
C2	0.7401 (2)	0.75033 (15)	0.26348 (16)	0.0210 (4)	
H2	0.7622	0.6904	0.2274	0.025*	
C3	0.83635 (18)	0.80073 (14)	0.35103 (16)	0.0190 (4)	
C4	0.81077 (18)	0.88872 (14)	0.40557 (15)	0.0172 (4)	
H4	0.8771	0.9213	0.4655	0.021*	
C4A	0.68395 (18)	0.92805 (13)	0.36933 (15)	0.0157 (3)	
C5	0.66897 (18)	1.09921 (13)	0.47909 (15)	0.0160 (3)	
H5	0.7573	1.1017	0.5261	0.019*	
C5A	0.62282 (17)	1.01868 (14)	0.40085 (14)	0.0152 (3)	
C6	0.58032 (18)	1.17500 (14)	0.48494 (15)	0.0177 (4)	
C7	0.44886 (19)	1.17234 (15)	0.41908 (16)	0.0196 (4)	
H7	0.3913	1.2259	0.4268	0.024*	
C8	0.40232 (18)	1.09231 (15)	0.34294 (16)	0.0193 (4)	
H8	0.3130	1.0893	0.2984	0.023*	
C8A	0.49060 (18)	1.01611 (14)	0.33353 (15)	0.0168 (4)	
C9A	0.58570 (18)	0.87634 (14)	0.28268 (15)	0.0175 (4)	
N9	0.46917 (15)	0.92922 (12)	0.26366 (13)	0.0185 (3)	
N10	−0.06092 (19)	0.97527 (16)	−0.28226 (17)	0.0359 (4)	
C10	0.34532 (18)	0.90394 (15)	0.17760 (16)	0.0208 (4)	
H10A	0.2718	0.9203	0.2125	0.025*	
H10B	0.3428	0.8299	0.1615	0.025*	
C11	0.32683 (18)	0.96151 (14)	0.06050 (16)	0.0189 (4)	
H11A	0.3385	1.0353	0.0771	0.023*	
H11B	0.3944	0.9395	0.0208	0.023*	
C12	0.19112 (18)	0.94248 (15)	−0.02221 (15)	0.0193 (4)	
H12A	0.1235	0.9614	0.0189	0.023*	
H12B	0.1810	0.8692	−0.0421	0.023*	
C13	0.17058 (19)	1.00466 (16)	−0.13739 (16)	0.0234 (4)	
H13A	0.1815	1.0778	−0.1169	0.028*	
H13B	0.2388	0.9857	−0.1778	0.028*	
C14	0.0405 (2)	0.98855 (16)	−0.21986 (17)	0.0247 (4)	

### Atomic displacement parameters (Å^2^)

**Table:**

	*U*^11^	*U*^22^	*U*^33^	*U*^12^	*U*^13^	*U*^23^
Br1	0.02541 (11)	0.02488 (11)	0.02659 (10)	0.00894 (8)	0.00780 (8)	−0.00022 (8)
Br2	0.02582 (11)	0.01562 (10)	0.02420 (10)	−0.00012 (7)	0.00630 (8)	−0.00316 (7)
C1	0.0282 (10)	0.0212 (10)	0.0139 (8)	−0.0040 (8)	0.0054 (7)	−0.0019 (7)
C2	0.0304 (11)	0.0171 (9)	0.0174 (9)	0.0008 (8)	0.0096 (8)	−0.0014 (7)
C3	0.0194 (9)	0.0205 (10)	0.0184 (9)	0.0034 (7)	0.0072 (7)	0.0033 (7)
C4	0.0198 (9)	0.0190 (9)	0.0131 (8)	−0.0001 (7)	0.0050 (7)	0.0014 (6)
C4A	0.0202 (9)	0.0164 (9)	0.0111 (8)	−0.0023 (7)	0.0051 (7)	0.0003 (6)
C5	0.0169 (9)	0.0167 (9)	0.0145 (8)	−0.0004 (7)	0.0046 (7)	0.0019 (6)
C5A	0.0165 (9)	0.0177 (9)	0.0123 (8)	−0.0001 (7)	0.0052 (7)	0.0034 (6)
C6	0.0241 (10)	0.0140 (9)	0.0155 (8)	−0.0021 (7)	0.0059 (7)	−0.0002 (6)
C7	0.0205 (9)	0.0193 (9)	0.0204 (9)	0.0054 (7)	0.0077 (7)	0.0052 (7)
C8	0.0167 (9)	0.0233 (10)	0.0173 (9)	0.0010 (7)	0.0033 (7)	0.0037 (7)
C8A	0.0195 (9)	0.0182 (9)	0.0124 (8)	−0.0013 (7)	0.0036 (7)	0.0030 (6)
C9A	0.0208 (9)	0.0195 (9)	0.0129 (8)	−0.0009 (7)	0.0054 (7)	0.0019 (6)
N9	0.0186 (8)	0.0212 (8)	0.0138 (7)	−0.0016 (6)	0.0010 (6)	−0.0002 (6)
N10	0.0297 (11)	0.0438 (12)	0.0288 (10)	−0.0051 (9)	−0.0018 (8)	0.0102 (8)
C10	0.0199 (10)	0.0229 (10)	0.0177 (9)	−0.0049 (7)	0.0015 (7)	0.0004 (7)
C11	0.0192 (9)	0.0196 (10)	0.0169 (8)	−0.0014 (7)	0.0030 (7)	0.0022 (7)
C12	0.0186 (9)	0.0216 (10)	0.0166 (8)	−0.0030 (7)	0.0029 (7)	0.0005 (7)
C13	0.0240 (10)	0.0257 (11)	0.0184 (9)	−0.0050 (8)	0.0017 (7)	0.0035 (7)
C14	0.0279 (11)	0.0258 (11)	0.0197 (9)	−0.0012 (8)	0.0048 (8)	0.0053 (8)

### Geometric parameters (Å, °)

**Table:**

Br1—C3	1.9033 (19)	C9A—C1	1.394 (3)
Br2—C6	1.9059 (18)	C9A—C4A	1.416 (2)
C1—H1	0.9500	N9—C8A	1.385 (2)
C2—C1	1.382 (3)	N9—C9A	1.380 (2)
C2—H2	0.9500	N9—C10	1.461 (2)
C3—C2	1.402 (3)	N10—C14	1.138 (3)
C4—C3	1.380 (3)	C10—H10A	0.9900
C4—H4	0.9500	C10—H10B	0.9900
C4A—C4	1.394 (3)	C11—C10	1.525 (2)
C4A—C5A	1.448 (2)	C11—H11A	0.9900
C5—C5A	1.397 (2)	C11—H11B	0.9900
C5—H5	0.9500	C12—C11	1.522 (3)
C5A—C8A	1.411 (3)	C12—H12A	0.9900
C6—C5	1.381 (3)	C12—H12B	0.9900
C6—C7	1.399 (3)	C13—C12	1.535 (2)
C7—H7	0.9500	C13—H13A	0.9900
C8—C7	1.380 (3)	C13—H13B	0.9900
C8—H8	0.9500	C14—C13	1.470 (3)
C8A—C8	1.393 (3)		
			
C2—C1—C9A	117.75 (18)	N9—C8A—C8	129.03 (17)
C2—C1—H1	121.1	C1—C9A—C4A	121.49 (17)
C9A—C1—H1	121.1	N9—C9A—C1	129.35 (18)
C1—C2—C3	120.53 (17)	N9—C9A—C4A	109.16 (16)
C1—C2—H2	119.7	C8A—N9—C10	124.59 (16)
C3—C2—H2	119.7	C9A—N9—C8A	108.68 (15)
C2—C3—Br1	118.28 (14)	C9A—N9—C10	126.55 (16)
C4—C3—Br1	119.19 (15)	N9—C10—C11	112.32 (15)
C4—C3—C2	122.51 (18)	N9—C10—H10A	109.1
C3—C4—C4A	117.46 (17)	N9—C10—H10B	109.1
C3—C4—H4	121.3	C11—C10—H10A	109.1
C4A—C4—H4	121.3	C11—C10—H10B	109.1
C4—C4A—C5A	133.33 (17)	H10A—C10—H10B	107.9
C4—C4A—C9A	120.22 (16)	C10—C11—H11A	109.4
C9A—C4A—C5A	106.44 (16)	C10—C11—H11B	109.4
C5A—C5—H5	121.5	C12—C11—C10	111.13 (15)
C6—C5—C5A	116.97 (17)	C12—C11—H11A	109.4
C6—C5—H5	121.5	C12—C11—H11B	109.4
C5—C5A—C4A	133.30 (17)	H11A—C11—H11B	108.0
C5—C5A—C8A	120.28 (16)	C11—C12—C13	110.92 (15)
C8A—C5A—C4A	106.41 (16)	C11—C12—H12A	109.5
C5—C6—Br2	119.25 (14)	C11—C12—H12B	109.5
C5—C6—C7	122.91 (17)	C13—C12—H12A	109.5
C7—C6—Br2	117.83 (14)	C13—C12—H12B	109.5
C6—C7—H7	119.8	H12A—C12—H12B	108.0
C8—C7—C6	120.35 (17)	C12—C13—H13A	109.1
C8—C7—H7	119.8	C12—C13—H13B	109.1
C7—C8—C8A	117.78 (17)	C14—C13—C12	112.69 (16)
C7—C8—H8	121.1	C14—C13—H13A	109.1
C8A—C8—H8	121.1	C14—C13—H13B	109.1
C8—C8A—C5A	121.68 (17)	H13A—C13—H13B	107.8
C1—C9A—C4A	121.49 (17)	N10—C14—C13	178.9 (2)
N9—C8A—C5A	109.28 (16)		
			
C3—C2—C1—C9A	1.4 (3)	N9—C8A—C8—C7	−179.45 (16)
Br1—C3—C2—C1	−179.17 (14)	C5A—C8A—C8—C7	1.3 (2)
C4—C3—C2—C1	−0.9 (3)	N9—C9A—C1—C2	179.02 (17)
C4A—C4—C3—Br1	177.36 (12)	C4A—C9A—C1—C2	−0.1 (3)
C4A—C4—C3—C2	−0.9 (3)	N9—C9A—C4A—C4	179.02 (15)
C5A—C4A—C4—C3	−176.78 (17)	N9—C9A—C4A—C5A	−1.80 (18)
C9A—C4A—C4—C3	2.1 (2)	C1—C9A—C4A—C4	−1.7 (3)
C4—C4A—C5A—C5	−0.1 (3)	C1—C9A—C4A—C5A	177.48 (15)
C4—C4A—C5A—C8A	−179.90 (18)	C9A—N9—C8A—C5A	−1.15 (19)
C9A—C4A—C5A—C5	−179.15 (17)	C9A—N9—C8A—C8	179.49 (17)
C9A—C4A—C5A—C8A	1.08 (18)	C10—N9—C8A—C5A	−176.60 (15)
C6—C5—C5A—C4A	179.22 (17)	C10—N9—C8A—C8	4.0 (3)
C6—C5—C5A—C8A	−1.0 (2)	C8A—N9—C9A—C1	−177.36 (17)
C4A—C5A—C8A—N9	0.02 (18)	C8A—N9—C9A—C4A	1.85 (19)
C4A—C5A—C8A—C8	179.44 (15)	C10—N9—C9A—C1	−2.0 (3)
C5—C5A—C8A—N9	−179.80 (14)	C10—N9—C9A—C4A	177.18 (15)
C5—C5A—C8A—C8	−0.4 (2)	C8A—N9—C10—C11	79.5 (2)
Br2—C6—C5—C5A	−179.64 (12)	C9A—N9—C10—C11	−95.1 (2)
C7—C6—C5—C5A	1.6 (2)	C12—C11—C10—N9	−173.89 (15)
Br2—C6—C7—C8	−179.52 (13)	C13—C12—C11—C10	177.23 (16)
C5—C6—C7—C8	−0.7 (3)	C14—C13—C12—C11	−179.95 (17)
C8A—C8—C7—C6	−0.7 (3)		
